# Early-stage alcohol-induced cardiomyopathy: not so much dilatation and dysfunction

**DOI:** 10.1093/ehjcr/ytad137

**Published:** 2023-03-30

**Authors:** Magdalena Carrillo-Bailen, Kaltoum ElMahraoui-ElGhazzaz, Jose Angel Urbano-Moral

**Affiliations:** Inherited Cardiac Conditions & Myocardial Diseases Unit, Cardiology Department, Jaen University Hospital, 10 Ejercito Espanol Ave., Jaen 23007, Spain; Inherited Cardiac Conditions & Myocardial Diseases Unit, Cardiology Department, Jaen University Hospital, 10 Ejercito Espanol Ave., Jaen 23007, Spain; Inherited Cardiac Conditions & Myocardial Diseases Unit, Cardiology Department, Jaen University Hospital, 10 Ejercito Espanol Ave., Jaen 23007, Spain

## Case report

**Figure 1:**
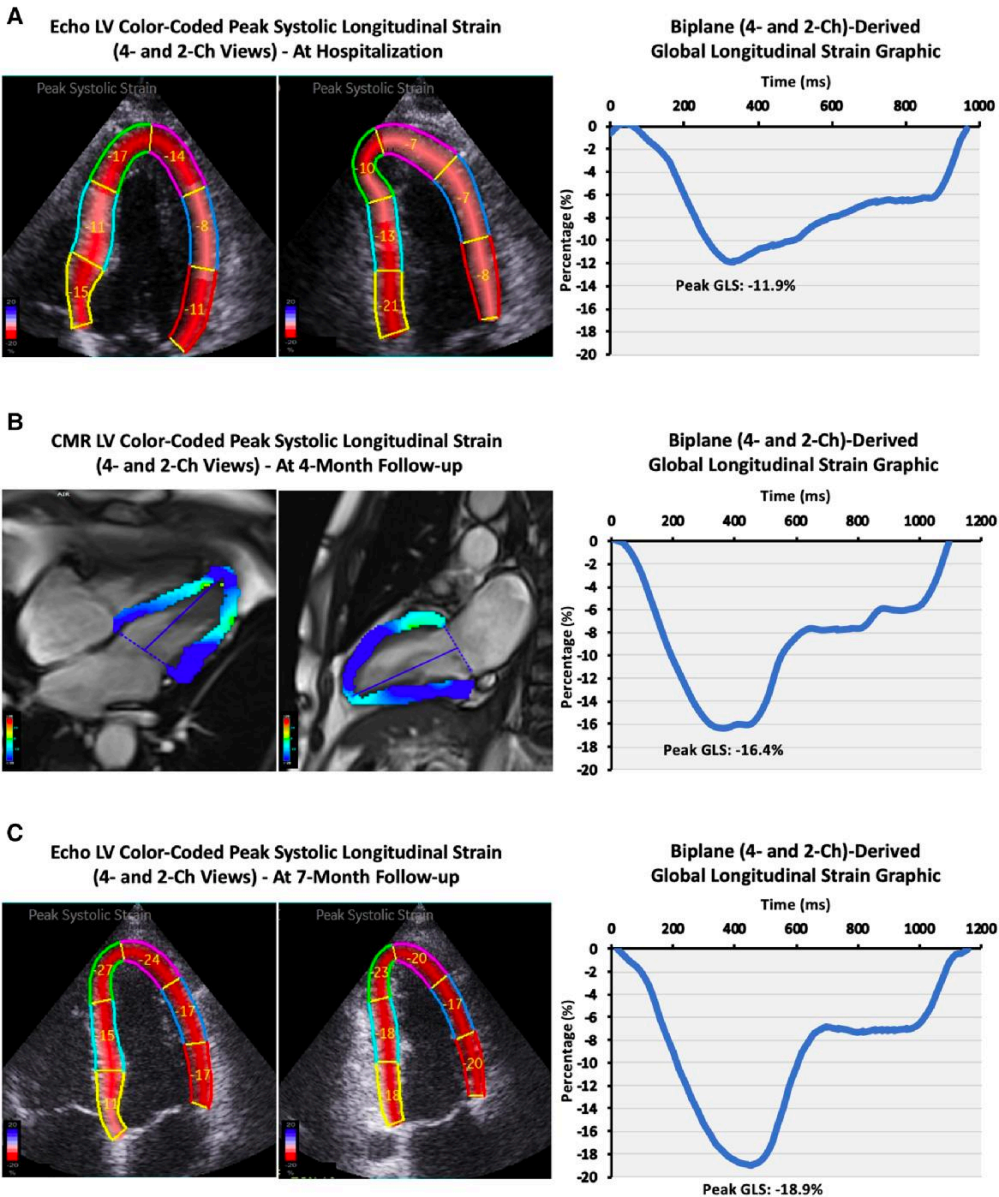
Left ventricular longitudinal strain patterns at hospitalization and during follow-up.

A 51-year-old male, smoker and drinker of five glasses of wine and three distilled spirits daily over 20 years, with no other cardiovascular risk factors, sustained a syncope and was transferred to the hospital. On arrival, he was asymptomatic, and his electrocardiogram showed sinus rhythm (85 b.p.m.) with no other abnormalities (see Supplementary material online, *Figure S1*). During his hospital stay, measurements of blood pressure, blood glucose, or lipids were all within the normal range. An echocardiogram demonstrated overall increased wall thickness (14 mm; see Supplementary material online, *Figure S1*) and left ventricular (LV) ejection fraction 57%; in contrast, myocardial deformation was significantly impaired (*Figure 1A*; see Supplementary material online, *Video S1*). Given the history of prior syncopes (×2), a coronary angiography was performed, which resulted in normal coronary arteries. Autoimmune conditions, Fabry disease, and amyloid light-chain or transthyretin amyloidosis were also ruled out. Eventually, alcohol abuse was suspected to be the likely cause of the LV morphofunctional alterations.

At 4-month follow-up, the patient remained asymptomatic, in sinus rhythm (80 b.p.m.), and confirmed complete cessation of alcohol consumption. A cardiovascular magnetic resonance demonstrated normal LV wall thickness and no late gadolinium enhancement, while LV deformation had experienced some improvement (*Figure 1B*). Seven months later, still in sinus rhythm (86 b.p.m.; see Supplementary material online, *Figure S1*) and without alcohol exposure, normalization of LV deformation parameters was demonstrated (*Figure 1C*; see Supplementary material online, *Video S2*). At this point, diagnosis of alcohol-induced cardiomyopathy was finally made.

Alcohol-induced cardiomyopathy is always a possibility in cases of significant long-standing alcohol intake, in the context of LV dilatation–dysfunction of no other origin evident. The case presented could account for an aborted course towards a more advanced LV remodelling status. Although it is not well understood how these changes evolve over time in the early stages, our case featured subclinical LV dysfunction, which progressively normalized after complete alcohol abstinence.

## Supplementary Material

ytad137_Supplementary_Data

## Data Availability

The data underlying this article will be shared on reasonable request to the corresponding author.

## Supplementary material

**Consent:** The authors confirm that written consent for submission and publication of this case report, including images and associated text, has been obtained from the patient in line with the Committee on Publication Ethics (COPE) guidelines.

**Funding:** None declared.

