# Supplementary figures and images for: Early-stage alcohol-induced cardiomyopathy: not so much dilatation and dysfunction
Source: Eur Heart J Case Rep. 2023 Mar 30;7(4):ytad137. doi: 10.1093/ehjcr/ytad137 (PMC10090477; doi:10.1093/ehjcr/ytad137)

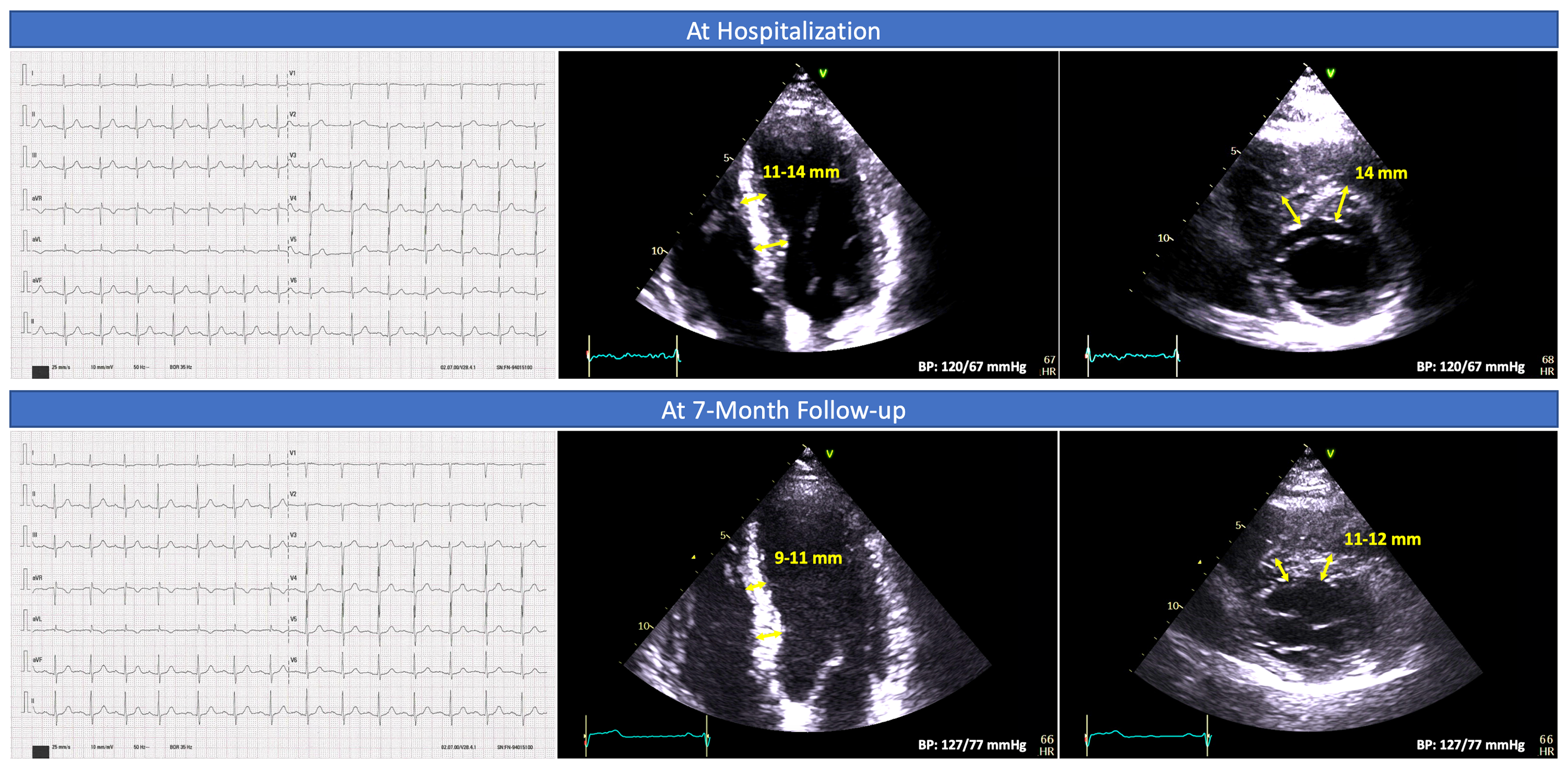

Supplement: ytad137_Supplementary_Data [file ytad137_supplementary_data.zip › Figure S1 (1).tiff]
